# Drugs targeting the NO-sGC-cGMP pathway in the treatment of patients with COPD-associated pulmonary hypertension: a systematic review

**DOI:** 10.3389/fphar.2025.1641932

**Published:** 2025-09-05

**Authors:** Abdullah A. Alqarni, Sara A. Alghamdi, Abdulelah M. Aldhahir, Jaber S. Alqahtani, Rayan A. Siraj, Ahmed H. Alasimi, Heba M. Bintalib, Abdulkareem A. AlGarni, Mansour Majrshi, Adel M. Alshabasy, Salma AlBahrani, Hassan Alwafi

**Affiliations:** ^1^ Department of Respiratory Therapy, Faculty of Medical Rehabilitation Sciences, King Abdulaziz University, Jeddah, Saudi Arabia; ^2^ Respiratory Therapy Unit, King Abdulaziz University Hospital, Jeddah, Saudi Arabia; ^3^ Respiratory Care Department, AlSalama Hospital, Jeddah, Saudi Arabia; ^4^ Respiratory Therapy Program, Department of Nursing, College of Nursing and Health Sciences, Jazan University, Jazan, Saudi Arabia; ^5^ Department of Respiratory Care, Prince Sultan Military College of Health Sciences, Dammam, Saudi Arabia; ^6^ Department of Respiratory Care, College of Applied Medical Sciences, King Faisal University, Al Ahsa, Saudi Arabia; ^7^ Department of Respiratory Therapy, Georgia State University, Atlanta, GA, United States; ^8^ Department of Respiratory Care, King Saud bin Abdulaziz University for Health Sciences, Jeddah, Saudi Arabia; ^9^ King Abdullah International Medical Research Centre, Jeddah, Saudi Arabia; ^10^ King Abdulaziz Hospital, The Ministry of National Guard Health Affairs, Al Ahsa, Saudi Arabia; ^11^ King Saud bin Abdulaziz University for Health Sciences, College of Applied Medical Sciences, Al Ahsa, Saudi Arabia; ^12^ National Heart and Lung Institute, Imperial College London, London, United Kingdom; ^13^ Respiratory Medicine, Royal Brompton Hospital, London, United Kingdom; ^14^ Anesthesia and Critical Care Department, King Abdulaziz University Hospital, Jeddah, Saudi Arabia; ^15^ Infectious Disease Unit, Specialty Internal Medicine, King Fahd Military Medical Complex, Dhahran, Saudi Arabia; ^16^ College of Medicine, Imam Abdulrahaman Bin Faisal University, Dammam, Saudi Arabia; ^17^ Department of Clinical Pharmacology and Toxicology, Faculty of Medicine, Umm Al-Qura University, Makkah, Saudi Arabia

**Keywords:** pulmonary hypertension, COPD, nitric oxide pathway, group 3PH, soluble guanylate cyclase stimulators, sGC stimulators, sildenafil, PDE5 inhibitors

## Abstract

**Background:**

Pulmonary hypertension (PH) due to chronic obstructive pulmonary disease (COPD) is categorized as group 3 PH and is associated with increased mortality and morbidity. Currently, there are no approved therapies for those who have PH secondary to COPD due to conflicting evidence. Therefore, this systematic review aims to summarize the current evidence on the effectiveness of drugs targeting the nitric oxide (NO)-soluble guanylate cyclase (sGC)-cyclic guanosine monophosphate (cGMP) pathway on clinical outcomes among patients with COPD-associated PH.

**Methods:**

We conducted a comprehensive search of electronic databases, including Embase, Medline, Cochrane, and Scopus, from inception to 1 February 2024. Studies investigating the efficacy of drugs targeting the NO-sGC-cGMP pathway on clinical outcomes in patients with COPD-associated PH were included. Exclusion criteria encompassed case reports, systematic reviews, review articles, conference abstracts with no full text, non-full-text articles, non-English manuscripts, opinion articles, and book chapters. Two distinct Cochrane risk-of-bias tools designed for randomized and non-randomized clinical trials were used to evaluate the risk of bias within the selected studies for inclusion.

**Results:**

Fourteen studies, comprising a total of 567 adult patients diagnosed with PH secondary to COPD, met the inclusion criteria and were included in this systematic review. Among these, nine studies reported significant improvements in clinical parameters related to pulmonary hemodynamics. Improvement in exercise capacity was observed in four out of seven studies. Three studies evaluated dyspnea severity and quality of life following treatment with agents targeting the NO-sGC-cGMP pathway. Of these, three demonstrated improvement in dyspnea severity while two reported enhancements in health-related quality of life. Substantial heterogeneity was evident regarding the potential of pharmacological agents targeting the NO-sGC-cGMP pathway to enhance gas exchange, lung function, and arterial oxygenation in COPD patients with concurrent PH.

**Conclusion:**

The short-term use of oral drugs targeting the NO-sGC-cGMP pathway, particularly sildenafil, demonstrates promising potential for enhancing pulmonary hemodynamics, exercise capacity, dyspnea severity, and health-related quality of life but not lung function and oxygenation status in adult patients with COPD-associated PH. Further double-blind, randomized, placebo-controlled trials are needed to assess the therapeutic benefits of agents targeting the NO-sGC-cGMP pathway, particularly inhaled therapies for managing PH due to COPD.

**Systematic Review Registration:**

https://www.crd.york.ac.uk/prospero/#recordDetails, CRD42023453503.

## 1 Introduction

Pulmonary hypertension (PH) is a hemodynamic state that is characterized by pulmonary vascular remodeling and vasoconstriction of pulmonary vessels, leading to sustained elevation in mean pulmonary arterial pressure (mPAP) (Hoeper et al., 2017; Humbert et al., 2013). The global prevalence of PH is around 1%, and it is estimated that PH affects 10% of patients above 65 years old (Hoeper et al., 2017; Wijeratne et al., 2018; Hoeper et al., 2016). PH is justified by multiple clinical conditions, which is why it is divided into five major categories according to its clinical relevance (Hoeper et al., 2017; Humbert et al., 2013; Simonneau et al., 2009). PH subgroups are: 1) pulmonary arterial hypertension (PAH), 2) PH due to left heart disease, 3) PH due to lung disease, 4) chronic thrombo-embolic PH, 5) PH with unclear and/or multifactorial mechanisms. Each of these categories is related to the underlying cause leading to pulmonary vasoconstriction and an increased mPAP.

Group 3 PH is the second most common group of all PH categories with the highest mortality rate (McGettrick and Peacock, 2020). It is developed mainly secondary to chronic obstructive pulmonary disease (COPD), hypoventilation syndrome, high altitude hypoxia, and interstitial lung disease (ILD). Among these conditions, COPD is the most common pulmonary disease associated with developing group 3 PH (Hoeper et al., 2017; Katiyar and Khare, 2018). Although the prevalence of PH in COPD is unclear due to the variations in the definition of PH and methods used for the diagnosis (Gologanu et al., 2013), previous studies have shown that the prevalence of PH in COPD patients ranges from 39.2% to 62.4% (Zhang et al., 2022; Gupta et al., 2018), with a greater prevalence in end-stage disease (Nathan et al., 2019). Several factors (e.g., cigarette smoke, hypoxia and inflammation) contribute to the development of PH in COPD patients (Karnati et al., 2021), which begins in the early stages of the disease through the remodeling of pulmonary arteries caused by the proliferation of smooth muscle cells leading to pulmonary vasoconstriction (Zakynthinos et al., 2011).

Generally, endothelial cells maintain the balance between vasodilators (nitric oxide (NO) and prostacyclin) and vasoconstrictors (thromboxane A_2_ (TXA_2_), and endothelin-1) mediators and prevent pulmonary artery cell proliferation. Endothelial dysfunction leads to a shift in the endothelium toward increased vasoconstrictor mediators and has been shown to be involved in the process of remodeling and constriction of pulmonary arteries (Alqarni et al., 2022). Although PH in COPD is incurable, and it is still unclear whether patients with PH in COPD may benefit from therapies targeting these dysfunctional pathways (NO, prostacyclin, and endothelin-1), research has been evaluating the effect of using targeted therapies approved for PAH in patients with PH due to COPD, with heterogeneous results. Among these three pathways, the NO pathway plays a vital role in the development of PH and is currently used as a therapeutic target for various forms of PH. The release of NO into vascular smooth muscle cells activates soluble Guanylyl Cyclase (sGC), which is an enzyme that converts Guanosine Triphosphate (GTP) into cyclic Guanosine Monophosphate (cGMP) (Evora et al., 2012). Consequently, this conversion through a series of reactions reduces intracellular calcium and, eventually, induces pulmonary artery vasodilation.

A disruption in the production of NO is a problem that causes persistent vasoconstriction and represents the rationale for the use of drugs targeting the NO-sGC-cGMP pathway for treating patients with COPD-associated PH. Given the fact that findings from studies assessing drugs NO-sGC-cGMP demonstrated promising results (Alkhayat and Eid, 2016; Li et al., 2021; Alp et al., 2006; Holverda et al., 2008; Vitulo et al., 2017; Karakitsos et al., 2013; Shrestha et al., 2017; Sharif-Kashani et al., 2014; Blanco et al., 2010; Blanco et al., 2013; Ghofrani et al., 2015; Pichl et al., 2019; Maron et al., 2022; Vonbank et al., 2003) and the use of inhaled sGC stimulator is currently under investigation for potential use (Merck and Dohme, 2023), we conducted this systematic review to summarize the currently available evidence on the effectiveness of drugs targeting NO-sGC-cGMP pathway on pulmonary hemodynamics, exercise capacity, dyspnea severity, oxygenation, and health-related quality of life among patients with COPD-associated PH.

## 2 Materials and methods

**FIGURE 1:**
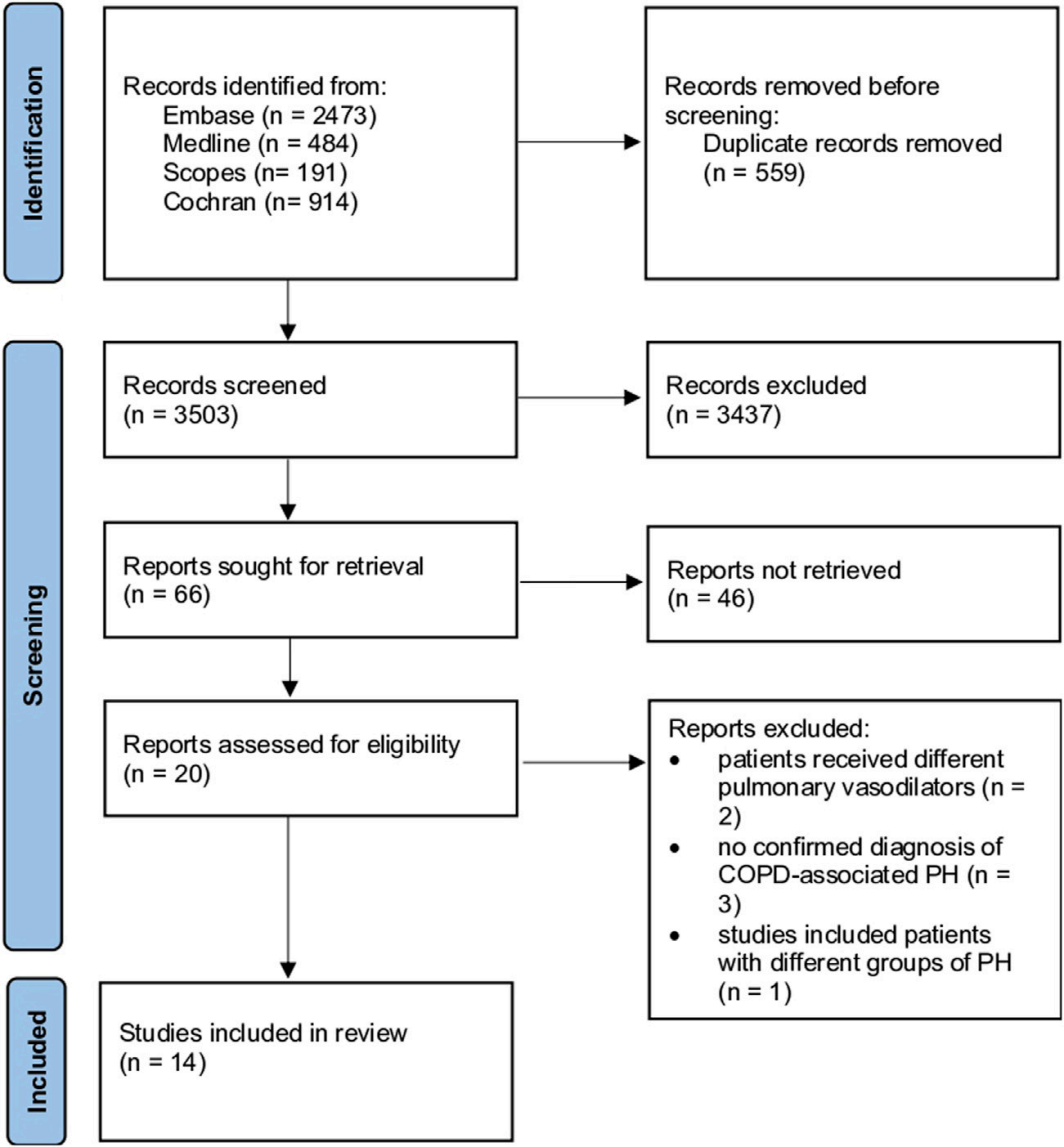
Flowchart illustrating a study selection process based on the Preferred Reporting Items for Systematic Review and Meta-Analysis Guidelines, including numbers of studies meeting eligibility criteria and numbers excluded. COPD: chronic obstructive pulmonary disease, PH: pulmonary hypertension.

The systematic review protocol was prospectively registered on PROSPERO (registration number: CRD42023453503). Studies retrieved were sent to EndNote and then entered into Rayyan software (https://www.rayyan.ai/), where blinding of the investigators was achieved. Abdullah A. Alqarni and SAA independently and blindly evaluated the titles and abstracts of all studies against the inclusion criteria. Disagreements between the two reviewers were resolved through discussion, and when consensus could not be reached, a third reviewer (AMA) was consulted to make the final decision. If the title and abstract were not informative enough, reviewers read the entire manuscript to determine whether the study should be included. In addition, we checked the references for further sources. We extracted the data following the Preferred Reporting Items for Systematic Reviews and Meta-Analyses (PRISMA) guidelines for the systematic review (Moher et al., 2015). A standardised data extraction sheet was used to extract data from suitable full-text articles. The extracted data are summarized and presented in Figure 1.

### 2.1 Data selection

We searched electronic databases of Embase, Medline, Cochrane, and Scopus from inception to 1 February 2024 for publications on drugs targeting the NO-sGC-cGMP pathway (including inhaled NO, riociguat, MK-5475, sildenafil, revatio, tadalafil, and adcirca) (see Supplementary Table S1). Studies that reported the findings of drugs targeting the NO-sGC-cGMP pathway on clinical outcomes among patients with PH due to COPD were included in this systematic review. We excluded case reports, systematic reviews, review articles, conference abstracts with no full text, non-full-text articles, non-English manuscripts, opinion articles, and book chapters. We did not specify a minimal study sample size for inclusion. To develop focused clinical questions, we used the PICO framework in our search strategy: P: population (adults with a confirmed diagnosis of COPD-associated PH), I: intervention (drugs targeting the NO-sGC-cGMP pathway), C: comparison (placebo or usual care), O: outcome (exercise capacity, pulmonary hemodynamics, lung function, severity of dyspnoea, quality of life, and oxygenation status).

### 2.2 Qualitative assessment of study methodology

We used two different Cochrane risk-of-bias tools designed for randomized and non-randomized clinical trials to evaluate the risk of bias within the selected studies in this review (see Supplementary Tables S1, S2). Two authors (Abdullah A. Alqarni and AMA) completed the assessment of study quality. For randomized clinical trials, we used the revised Cochrane risk-of-bias tool (Sterne et al., 2019), which consists of six domains: risk of bias 1) arising from the randomization process; 2) bias due to deviations from the intended interventions (assignment to intervention); 3) bias due to deviations from the intended interventions (adhering to intervention); 4) bias due to missing outcome data; 5) bias in the measurement of the outcome; 6) bias in the selection of the reported result. For non-randomized clinical trials, we used the Cochrane risk of bias in the non-randomized studies assessment (Sterne et al., 2016). The tool consists of seven domains, which focus on 1) bias due to confounding; 2) bias in the selection of participants for the study; 3) bias in the classification of interventions; 4) bias due to deviations from intended interventions; 5) bias due to missing data; 6) bias in the measurement of outcomes; 7) bias in the selection of the reported result. Several questions were answered under each domain before the risk of bias in each study was classified into low, medium, or high based on the total score. The randomized clinical trial was judged to be at high risk of bias if at least one domain was at high risk of bias, or some concerns have been reported in multiple domains of the tool. The study is judged to have some concerns in at least one domain for this result, without having any domain at high risk of bias. The study was judged to be at low risk of bias if all domains were classified to have a low risk of bias. For non-randomized studies, the risk of bias for each study was considered low if all domains were classified as having a low risk of bias. The study was reported to have a at medium risk of bias only if at least one domain was at medium risk of bias and the remaining domains at low risk of bias. The study is judged to be at serious risk of bias in at least one domain but not at critical risk of bias in any domain. The study is judged to be at critical risk of bias in at least one domain.

## 3 Results

### 3.1 Description of the included studies

Table 1 presents the summary characteristics of the studies included in this systematic review. Fourteen studies involving a total of 567 COPD patients with PH were included in this systematic review to assess the clinical effectiveness of drugs targeting the NO-sGC-cGMP pathway, namely, sildenafil (Alkhayat and Eid, 2016; Li et al., 2021; Alp et al., 2006; Holverda et al., 2008; Vitulo et al., 2017; Karakitsos et al., 2013; Shrestha et al., 2017; Sharif-Kashani et al., 2014; Blanco et al., 2010; Blanco et al., 2013), riociguat (Ghofrani et al., 2015; Pichl et al., 2019), tadalafil (Maron et al., 2022), and inhaled NO (Vonbank et al., 2003). Among these studies, seven were randomized controlled trials (Vitulo et al., 2017; Shrestha et al., 2017; Sharif-Kashani et al., 2014; Blanco et al., 2010; Blanco et al., 2013; Maron et al., 2022; Vonbank et al., 2003), five were non-randomized controlled trials (Alkhayat and Eid, 2016; Li et al., 2021; Alp et al., 2006; Holverda et al., 2008; Ghofrani et al., 2015), one was a non-randomized non-controlled trial (Karakitsos et al., 2013), and one was a retrospective analysis study (Pichl et al., 2019). The clinical outcomes evaluated in most of the included studies in this systematic review were exercise capacity, pulmonary hemodynamics, lung function, severity of dyspnoea, quality of life, and oxygenation status.

**TABLE 1:** Summary of Studies on drugs targeting the NO-sGC-cGMP pathway in the treatment of patients with COPD-associated pulmonary hypertension.

Authors, years of publication & country	Study design	COPD severity: n (male)	Medication, (dose, duration, groups)	Diagnostic test to confirm PH, mean/median mPAP or PASP	Baseline PVR, CO, and CI	Outcome	Findings
Maron et al. (2022), United States	Prospective, multicenter, placebo‐controlled randomized clinical trial	GOLD 1-4: 24 (24)	Group A Control: Placebo Group B Treated: Oral tadalafil (40 mg) given once-daily for 12 months	RHC confirmed PH Control group: baseline median (IQR) mPAP 33 (27–35) Treated group: baseline median (IQR) mPAP 30 (27–34)	PVR: Group A control 4.5 [2.8–5.9] WU, Group B treated 3.9 [3.1–5.5] WU CO: Group A control 4.5 [2.8–5.9] L/min, Group B treated 3.9 [3.1–5.5] L/min CI: N/A	Primary: Exercise capacity compared to baseline at 12 months Secondary: Pulmonary hemodynamics, exercise capacity, dyspnea, health-related quality of life	Primary: No significant difference in exercise capacity between groups at 12 months (6MWD p = 0.65) Secondary: Tadalafil improved dyspnea (UCSD SOBQ p = 0.02) and health-related quality of life (SGRQ p = 0.049). No significant differences in pulmonary hemodynamics, 6MWD, and quality of life
Li et al. (2021), China	Non-randomized controlled clinical trial	GOLD N/A: 90 (47)	Group A: Sildenafil (50 mg) + Bosentan (62.5 mg) both twice a day for 4 weeks Group B: Iloprost (5 μg/dose) + Bosentan for 4 weeks	Doppler ultrasound confirmed PH	PVR: N/A, CO: N/A, CI: N/A	Primary: Change in exercise capacity, pulmonary function, oxygenation, inflammatory mediators, and cardiac function compared to Group B	Sildenafil group showed significantly higher 6MWD, improved FEV1, FVC, and PaO_2_ (p < 0.05). Lower inflammatory cytokine levels (IL-17, IL-13, CRP) in sildenafil group (p < 0.05). Significant improvement in RVEF (p < 0.05)
Pichl et al. (2019), Germany	Retrospective analysis	GOLD 4: 7 (0)	One group: Riociguat	RHC confirmed PH, mean mPAP 46 mmHg	PVR: mean ± SEM 681 ± 143 dyn·cm−2·s−1 CI: 2.25 ± 0.16 mL·min−1·kg−1	Primary: Pulmonary hemodynamics, exercise capacity, pulmonary function, and oxygenation compared with baseline	Pulmonary hemodynamics: No significant change in mPAP (46 vs 38 mmHg, p = 1.0) and CI (2.25 vs 3.02 mL·min−1·kg−1, p = 1.0) Significant change in PVR (681 vs 389 dyn·cm−2·s−1, p < 0.001) No significant difference in exercise capacity (6MWT), pulmonary function (FEV1, DLCO), or oxygenation (PaO_2_)
Vitulo et al. (2017), Italy	16-week, double-blind, multicenter, randomized, placebo-controlled trial	GOLD 2 and 3: 28 (21)	Control: Placebo Treated: Sildenafil (20 mg) 3 times a day for 16 weeks	RHC confirmed PH, mean mPAP 39 mmHg	PVR: Control 6.3 ± 3.1 WU, Treated 7.00 ± 2.6 WU CO: N/A CI: N/A	Primary: Pulmonary hemodynamics compared with baseline Secondary: Changes in exercise capacity, pulmonary function, oxygenation, dyspnea, and quality of life	Primary: Significant decrease in PVR in treated group (7.01 to 5.72 WU, p = 0.04) Secondary: No significant differences in exercise capacity (6MWT), pulmonary function (spirometry), oxygenation (PaO_2_) Improvement in DLCO% (p = 0.04), dyspnea (mMRC p = 0.03), and quality of life (SF36 p = 0.04) in treated group
Shrestha et al. (2017), Nepal	Prospective randomized controlled trial in two tertiary centers	GOLD 1-3: 61 (28)	Group A Control: standard treatment Group B Treated: Sildenafil (25 mg) 3 times a day for 4 weeks	Echocardiography confirmed PH Control group: Baseline mean ± SD of PASP was 75.9 ± 17.89 mmHg Treated group: Baseline mean ± SD of PASP was 66.77 ± 11.61 mmHg	PVR: N/A CO: N/A CI: N/A	Primary: Pulmonary hemodynamics and exercise capacity compared to baseline Secondary: Changes in dyspnea and PH severity	Primary: Significant decrease in PASP in treated group (p = 0.048). Significant improvement in 6MWT in treated group (p = 0.047) Secondary: Significant improvement in mMRC (p = 0.037). No significant difference in WHO functional class (p = 0.071). No significant difference in modified Borg scale (p = 0.401)
Alkhayat and Eid (2016), Egypt	Prospective placebo-controlled trial	GOLD 1-4: 139 (106), Treated: 69 (49), Control: 70 (57)	Treated group: Sildenafil (20mg, 3x/day for 12 weeks) + conventional therapy Control group: Placebo + conventional therapy	Echocardiography Control group: Baseline mean ± SD of PAP was 56 ± 16 mmHg Treated group: Baseline mean +SD of PAP was 45 ± 13 mmHg	PVR: N/A CO: N/A, CI: N/A	Primary: Exercise capacity Secondary: Pulmonary hemodynamics	Primary: Sildenafil significantly improved exercise capacity (6MWT difference: 51m, p < 0.001) Secondary: mPAP reduction: Control: +0.6 mmHg, Treated: -2.1 mmHg (p < 0.05)
Sharif-Kashani et al. (2014), Iran	Prospective, randomized, open-label parallel group study	GOLD N/A: 40 (31)	Treated group: Sildenafil (20-25mg, 2x/day for 2 weeks) Control group: Amlodipine (2.5-7.5mg, 1x/day for 2 weeks)	Echocardiography Control group: Baseline mean ± SD of PASP was 58.0 ± 11.8 mmHg Treated group: Baseline mean +SD of PASP was 63.0 ± 12.5 mmHg	PVR: N/A, CO: N/A, CI: N/A	Primary: NT-proBNP levels Secondary: Pulmonary hemodynamics	Primary: No significant difference in NT-proBNP levels (p = 0.185) Secondary: No significant difference in PASP (p = 0.164)
Ghofrani et al. (2015), Germany	Exploratory, non-randomized, non-blinded, non-controlled pilot study	GOLD 2-3: 22 (11)	Riociguat (1 or 2.5 mg, single dose)	Cardiac catheterization (mPAP ≥ 23 mmHg)	PVR: Riociguat 1mg: 353, 2.5mg: 370 dyn.s.cm-5 CO: 1mg: 4.94, 2.5mg: 4.63 L/min, CI: N/A	Primary: Hemodynamic parameters Secondary: Lung function & gas exchange	Primary: mPAP: 1mg: -3.60 ± 3.41, p = 0.0086, 2.5mg: -4.83 ± 4.17, p = 0.0020 Secondary: No significant changes in lung function (p > 0.05)
Blanco et al. (2013), Spain	Double-blind, randomized, placebo-controlled trial	GOLD 3: 60 (54)	Treated group: Sildenafil (20mg, 3x/day for 3 months) + pulmonary rehabilitation Control group: Placebo + pulmonary rehabilitation	Right heart catheterization (mPAP ≥ 25 mmHg)	PVR: N/A, CO: N/A, CI: N/A	Primary: Cycle endurance time Secondary: 6MWT, oxygen uptake, quality of life	Primary: Sildenafil improved cycle endurance time (149s vs 169s on Placebo, p = 0.05) Secondary: No significant changes in 6MWT or oxygen uptake (p > 0.05)
Karakitsos et al. (2013), Greece	Prospective, non-randomized, non-controlled, single arm interventional trial	GOLD 1-4: 12 (9)	Sildenafil (80mg/day for 3 days) after dobutamine administration	RHC (mPAP: 49, 95% CI: 44-53 mmHg)	PVR: 844 ± 115 dynes.s.cm-5/m2, CO: 2.72 ± 0.22 L/min/m2	Primary: Pulmonary hemodynamics Secondary: Oxygenation	Primary: mPAP: -19% (p < 0.01), PVR: -51% (p < 0.01), CI: +54% (p < 0.01) Secondary: PaO_2_/FiO_2_ improvement (p < 0.05)
Blanco et al. (2010), Spain	Randomized, open label, with blind evaluation, dose comparison trial	GOLD 3 and 4: 20 (17)	Group A: Sildenafil 20 mg (n=11) twice daily for 2 weeks Group B: Sildenafil 40 mg (n=9) twice daily for 2 weeks	Doppler echocardiography, Right heart catheterization (mean PAP >20 mmHg at rest)	PVR: 456 ± 191 dyn.s.cm-5 CO: 4.90 ± 0.95 L/min	Primary: Sildenafil effect on pulmonary hemodynamics & gas exchange at rest and during exercise	mPAP: Decrease of 6 mmHg at rest (p < 0.05) PVR: Decrease of 110 dyn.s.cm-5 (p < 0.05) PaO2: Decrease of 6 mmHg (p < 0.05) at rest mPAP: Decrease of 11 mmHg during exercise (p < 0.05) PVR: Decrease of 136 dyn.s.cm-5 (p < 0.05) PaO2: Decrease of 1 mmHg during exercise (p>0.05)
Holverda et al. (2008), Amsterdam	Prospective clinical trial	GOLD 2-4: 18 (11)	Sildenafil 50 mg oral single dose & Placebo	Right heart catheterization (mPAP ≥ 25 mmHg at rest, ≥ 30 mmHg during exercise)	PVR: 280 ± 180 dyn.s.cm-5 (rest), 319 ± 209 dyn.s.cm-5 (exercise) CO: 5.5 ± 1.0 L/min (rest), 8.0 ± 1.7 L/min (exercise)	Primary: Acute effects of Sildenafil on mPAP, PVR, CO at rest and during exercise Secondary: Maximal exercise capacity	Primary: mPAP: Decreased from 23 ± 10 to 20 ± 10 at rest, 35 ± 14 to 30 ± 14 during exercise (p < 0.05) PVR: Decreased from 280 ± 180 to 251 ± 217 at rest, 319 ± 209 to 314 ± 288 during exercise CO: Increased from 5.5 ± 1.0 to 6.1 ± 1.7 at rest, no change during exercise Secondary: Maximal exercise test: No change
Alp et al. (2006), Germany	Prospective clinical trial	GOLD 3-4: 6 (4)	Sildenafil 50 mg oral twice daily for 3 months	Echocardiography for PH diagnosis	PVR: 401 ± 108 dyn.s.cm-5	Primary: Pulmonary hemodynamics Secondary: Exercise capacity	Primary: mPAP: Decreased from 30.2 ± 5.5 mmHg to 24.6 ± 4.2 mmHg (p < 0.05) PVR: Decreased from 401 ± 108 dyn.s.cm-5 to 264 ± 52 dyn.s.cm-5 (p < 0.05) Secondary: Exercise capacity: Increased from 351 ± 49 m to 433 ± 52 m (p < 0.05)
Vonbank et al. (2003), Vienna	Randomized controlled prospective open study	GOLD 3: 40 (27)	Oxygen alone (n=20) vs Oxygen + NO (15-25 ppm, n=20) for 3 months	Right heart catheterization (mPAP ≥ 25 mmHg)	PVR: 259 ± 101.7 dyne.s-1.cm-5 (oxygen) 276.9 ± 96.6 dyne.s-1.cm-5 (oxygen + NO)	Primary: Effect of oxygen + NO on PVR compared to oxygen alone after 3 months	mPAP decreased by 7 mmHg (p < 0.001) PVR decreased by 103.8 dyne.s-1.cm-5 (p < 0.001), CO increased by 0.5 L/min (p < 0.025), CI increased by 0.4 L/min/m2 PaO2: No change FEV1: No change

**Abbreviations:** PH, pulmonary hypertension; COPD, Chronic Obstructive Lung Disease; GOLD2, Global Initiative for Chronic Obstructive Lung Disease 2 (moderate COPD) GOLD3, Global Initiative for Chronic Obstructive Lung Disease 2 (severe COPD); PGI2, prostaglandin I2; mPAP, mean pulmonary artery pressure; RHC, right heart catheterization; ABG, arterial blood gas; WHO-FC, World Health Organization functional class; PaO2, partial pressure of oxygen; 6MWT, 6-minute walk test; PFT, pulmonary function tests; SGRQ, St George's respiratory questionnaire; FEV1, forced expiratory volume in one second; FVC, forced vital capacity; VE, minute ventilation; DLCO, diffusing capacity of the lungs for carbon monoxide; QOL, quality of life; PaCO2, partial pressure of carbon dioxide in arterial blood; SaO2, oxygen saturation of arterial blood; DA–a O2, alveolar-arterial oxygen gradient; VE/VO2, ventilatory equivalent for oxygen; VE/VCO2, ventilatory equivalent for carbon dioxide; Qs/Qt, pulmonary shunt fraction; PVR, pulmonary vascular resistance; PAWP, pulmonary arterial wedge pressure; RAP, right atrial pressure; CIx, cardiac index; CO, cardiac output; MAP, mean arterial pressure; SD, standard deviation.

### 3.2 Exercise capacity

Seven studies (Alkhayat and Eid, 2016; Li et al., 2021; Alp et al., 2006; Vitulo et al., 2017; Shrestha et al., 2017; Pichl et al., 2019; Maron et al., 2022) evaluated whether medications targeting the NO-sGC-cGMP pathway can improve exercise capacity (assessed as a 6-min walk test (6MWT)) in COPD patients with PH. Among these studies, three reported no significant improvement in exercise capacity (Vitulo et al., 2017; Pichl et al., 2019; Maron et al., 2022). For instance, it has been reported that tadalafil and riociguat showed no significant effect on exercise capacity (Pichl et al., 2019; Maron et al., 2022). In a randomized controlled multicenter clinical trial, sildenafil failed to improve exercise capacity among patients with severe COPD-associated PH (Vitulo et al., 2017). On the other hand, four studies showed a significant improvement in exercise capacity in response to sildenafil use compared with baseline (Alkhayat and Eid, 2016; Li et al., 2021; Alp et al., 2006; Shrestha et al., 2017). A prospective clinical trial by Alp et al. revealed that sildenafil significantly increased the 6MWT distance from 351 ± 49 m to 433 ± 52 m after 3 months (Alp et al., 2006). Other clinical trials have also reported that sildenafil enhanced exercise capacity in COPD patients with PH (Alkhayat and Eid, 2016; Li et al., 2021; Shrestha et al., 2017). Taken together, four out seven studies demonstrated an improvement in exercise capacity in response to pharmacological interventions targeting the NO-sGC-cGMP pathway—particularly sildenafil—in patients with COPD-associated PH.

### 3.3 Pulmonary hemodynamics

This systematic review examined pulmonary hemodynamic parameters in 12 studies (Alkhayat and Eid, 2016; Alp et al., 2006; Holverda et al., 2008; Vitulo et al., 2017; Karakitsos et al., 2013; Shrestha et al., 2017; Sharif-Kashani et al., 2014; Blanco et al., 2010; Ghofrani et al., 2015; Pichl et al., 2019; Maron et al., 2022; Vonbank et al., 2003). Among these, three studies demonstrated that sildenafil, tadalafil, and riociguat did not cause a significant difference in pulmonary hemodynamic parameters (e.g., mPAP, pulmonary vascular resistance (PVR), cardiac index (CI), and pulmonary artery wedge pressure (PAWP)) between control and treated groups (Sharif-Kashani et al., 2014; Pichl et al., 2019; Maron et al., 2022). Conversely, a prospective, non-randomized, non-controlled trial conducted by Karakitsos et al. demonstrated that sildenafil was significantly associated with reductions in mPAP and PVR (Karakitsos et al., 2013). Moreover, an exploratory pilot study by Ghofrani et al. reported that riociguat administration was preceded by short-term administration of inhaled NO, resulted in decreased mPAP and PVR alongside improvements in cardiac output (Ghofrani et al., 2015). Similar enhancements have also been reported in cardiac function and significant reductions in mPAP, PVR, and PAWP among COPD patients with PH (Alkhayat and Eid, 2016; Alp et al., 2006; Holverda et al., 2008; Vitulo et al., 2017; Shrestha et al., 2017; Blanco et al., 2010; Vonbank et al., 2003). Collectively, nine out of twelve studies demonstrated significant improvements in pulmonary hemodynamic parameters following treatment with agents targeting the NO-sGC-cGMP pathway.

### 3.4 Lung function

Five studies in this review assessed whether medications targeting the NO-sGC-cGMP pathway could improve spirometry and diffusion capacity parameters among COPD patients with PH (Li et al., 2021; Vitulo et al., 2017; Ghofrani et al., 2015; Pichl et al., 2019; Vonbank et al., 2003). No statistically significant differences were observed in forced expiratory volume in 1 s (FEV_1_) or diffusing capacity of the lung for carbon monoxide (DLCO) after the use of riociguat either alone (Pichl et al., 2019) or after short-term administration of inhaled NO in COPD patients with PH (Ghofrani et al., 2015). Similarly, sildenafil did not significantly improve spirometry parameters, although an increase in DLCO was reported (Vitulo et al., 2017). Conversely, a non-randomized controlled clinical trial reported sildenafil enhanced spirometry parameters, including FEV_1_, forced vital capacity (FVC), and the FEV_1_/FVC ratio, compared with inhaled iloprost (Li et al., 2021). Interestingly, long-term use of inhaled NO significantly reduced FEV_1_ and the FEV1/FVC ratio in a randomized controlled prospective open study conducted by Vonbank et al. among 40 patients with severe COPD (Vonbank et al., 2003). Taken together, two out of five studies reported improvements in lung function following the use of drugs targeting the NO-sGC-cGMP pathway in patients with COPD-associated PH.

### 3.5 Severity of dyspnoea

Data concerning the severity of dyspnea were documented as a secondary outcome in three studies, encompassing a collective cohort of 113 patients diagnosed with COPD-associated PH (Vitulo et al., 2017; Shrestha et al., 2017; Maron et al., 2022). In a prospective randomized controlled trial conducted by Shrestha et al., oral sildenafil significantly improved dyspnea severity as measured by the modified Medical Research Council (mMRC) scale at week four. However, no significant change was observed when assessed using the modified Borg scale (Shrestha et al., 2017). Similarly, the mMRC score (but not the modified Borg scale) was significantly improved in response to sildenafil use (Vitulo et al., 2017). Additionally, in a prospective, multicenter, placebo‐controlled randomized controlled trial conducted by Maron et al., the daily administration of a single oral dose of 40 mg tadalafil was associated with a significant improvement in dyspnea severity at 6 months among 24 patients presenting with mild to very severe COPD in conjunction with PH (Maron et al., 2022). Collectively, all three studies that evaluated the impact of drugs targeting the NO-sGC-cGMP pathway reported improvements in dyspnea severity.

### 3.6 Quality of life

A total of three studies have assessed quality of life following treatment with drugs targeting the NO-sGC-cGMP pathway (Vitulo et al., 2017; Blanco et al., 2013; Maron et al., 2022). In a double-blinded randomized controlled trial conducted by Blanco et al., involving 60 patients with severe COPD, no statistically significant changes in quality of life were found after receiving 20 mg of sildenafil three times daily for 3 months (Blanco et al., 2013). Contrarily, Vitulo et al. reported significant improvements in quality of life following similar doses of sildenafil administered for 16 weeks (Vitulo et al., 2017). Furthermore, a prospective, multicenter, placebo-controlled randomized controlled trial by Maron et al. demonstrated that oral tadalafil administered at a dose of 40 mg once daily for 6 months significantly enhanced health-related quality of life among COPD patients with PH (Maron et al., 2022). Taken together, two out of three studies demonstrated improvements in health-related quality of life following treatment with agents targeting the NO-sGC-cGMP pathway.

### 3.7 Oxygenation

Eight studies included in this review looked at the partial pressure of oxygen (PaO_2_) and PaO_2_/FiO_2_ ratios as clinical outcomes for monitoring arterial oxygenation among COPD patients with PH (Li et al., 2021; Vitulo et al., 2017; Karakitsos et al., 2013; Blanco et al., 2010; Blanco et al., 2013; Ghofrani et al., 2015; Pichl et al., 2019; Vonbank et al., 2003). A randomized controlled trial demonstrated that the use of sildenafil adversely impacted arterial oxygenation, leading to a reduction in PaO_2_ by −6 mm Hg at rest. However, this effect was not statistically significant during exercise following sildenafil administration (Blanco et al., 2010). Furthermore, no significant difference was reported in the oxygenation parameters after 2 h of riociguat administration in patients with moderate to severe COPD and PH (Ghofrani et al., 2015). In addition, the findings of the three studies demonstrated no significant differences in arterial oxygenation, as assessed by PaO_2_ following administration of sildenafil (Vitulo et al., 2017; Blanco et al., 2013) and riociguat (Pichl et al., 2019). A randomized controlled prospective open study by Vonbank et al. indicated adverse effects of long-term inhaled NO administration on oxygenation status, resulting in a significant decrease in PaO_2_ compared to baseline (Vonbank et al., 2003). In contrast, a non-randomized controlled trial involving 90 COPD patients demonstrated a significant improvement in PaO_2_ levels among those receiving oral sildenafil compared with those receiving inhaled iloprost (Li et al., 2021). Likewise, Karakitsos et al. reported significant changes in the PaO_2_/FiO_2_ ratio, which increased markedly from 52% to 86% following the administration of sildenafil via nasogastric tube for 3 days after dobutamine administration (Karakitsos et al., 2013). Collectively, only two out of the eight studies included in this review reported improvements in oxygenation status following treatment with drugs targeting the NO-sGC-cGMP pathway.

## 4 Discussion

To our knowledge, this is the first systematic review to summarize the currently available evidence on the effectiveness of agents targeting the NO-sGC-cGMP pathway on clinical outcomes in patients with COPD-associated PH. Although our review delineated notable heterogeneity concerning the potential of such agents to augment lung function and arterial oxygenation, discernible improvements were documented in pulmonary hemodynamics, exercise capacity, and dyspnea severity, thereby positing a potential enhancement in health-related quality of life in those with COPD-associated PH. These observations provide insight into the potential therapeutic benefits of drugs targeting NO-sGC-cGMP and point to the unmet need for a large randomized controlled trial to explore further the efficacy of using therapies, particularly inhaled therapies targeting NO-sGC-cGMP pathway for the management of PH due to COPD.

The 6MWT is the most widely used measure for assessing exercise capacity in pulmonary hypertension (PH). This systematic review suggests that sildenafil, unlike tadalafil and riociguat, can enhance exercise capacity in mild to moderate COPD-associated PH (Alkhayat and Eid, 2016; Li et al., 2021; Alp et al., 2006; Vitulo et al., 2017; Shrestha et al., 2017; Pichl et al., 2019; Maron et al., 2022). The differential effects of these therapies may be attributed to their pharmacological properties, with sildenafil’s quicker onset of action potentially providing more immediate benefits, especially in less severe cases (Croom and Curran, 2008). The impact of other targeted PH therapies on exercise capacity in COPD-associated PH remains unclear. Our previous review found that only three studies evaluated the effect of inhaled prostaglandin I_2_ analogues on exercise capacity in this population (Alqarni et al., 2023a), with one study reporting significant improvement in the 6MWT (Dernaika et al., 2010), while the other two found no effect (Bajwa et al., 2017; Boeck et al., 2012). A current 24-week randomized, double-blind, placebo-controlled clinical trial is investigating the efficacy and safety of an inhaled sGC stimulator in improving exercise capacity for adults with COPD-related PH (Merck and Dohme, 2023). This ongoing trial holds promise for advancing the management of patients with group 3 PH, an area where previous trials have been insufficient.

PH is characterized by an elevated mPAP of ≥20 mmHg at rest, often accompanied by increased PVR, leading to progressive remodeling of the pulmonary vasculature and potential right heart failure (Montani et al., 2013). Our systematic review highlights a broad consensus on the effectiveness of selective vasodilatory agents in improving pulmonary hemodynamics. While some studies reported no significant effects, likely due to small sample sizes and short trial durations (Sharif-Kashani et al., 2014; Pichl et al., 2019; Maron et al., 2022), several randomized controlled trials (RCTs) have demonstrated notable improvements in pulmonary hemodynamics and clinical outcomes (Alkhayat and Eid, 2016; Alp et al., 2006; Holverda et al., 2008; Vitulo et al., 2017; Karakitsos et al., 2013; Shrestha et al., 2017; Blanco et al., 2010; Vonbank et al., 2003). Ghofrani et al. found that both 1 mg and 2.5 mg doses of riociguat significantly reduced mPAP and PVR while enhancing cardiac output (Ghofrani et al., 2015). Similarly, studies on sildenafil and tadalafil have shown reductions in mPAP, as assessed by echocardiography, in patients with severe COPD (Rao et al., 2011; Goudie et al., 2014). Non-clinical studies further support these findings, with sildenafil demonstrating reduced mPAP and vascular remodeling in hypoxia-induced PH models (Sebkhi et al., 2003), and inhaled riociguat lowering PVR in a high-fidelity pulmonary simulator from COPD-associated PH patients (Saffaran et al., 2018). Despite these promising results, no disease-specific therapy has been approved for COPD-associated PH, as current management remains focused on treating the underlying disease per international guidelines (Galie et al., 2015). This is likely due to the absence of large clinical trials assessing the long-term impact of NO-sGC-cGMP-targeting agents on pulmonary hemodynamics in this population.

Spirometry remains the primary tool for assessing lung function, with diffusing capacity for carbon monoxide (DLCO) serving as a key measure for monitoring treatment efficacy in chronic lung diseases, particularly COPD (Humbert et al., 2022; Blanco et al., 2020). However, evidence supporting the role of NO-targeted therapy in improving lung function in COPD patients with PH remains limited. Clinical trials have largely failed to show significant improvements in lung function indices with pulmonary vasodilators, including sildenafil (Vitulo et al., 2017) and riociguat, whether used alone (Pichl et al., 2019) or following inhaled NO administration (Ghofrani et al., 2015). Moreover, long-term inhaled NO with oxygen has been linked to worsening lung function in this population (Vonbank et al., 2003). Given the already severe impairment in lung function among COPD patients, it is unsurprising that riociguat, sildenafil, and inhaled NO have shown little benefit in this regard. Interestingly, a combination of bosentan (an endothelin receptor antagonist) and sildenafil resulted in significant improvements in spirometry parameters, including FEV1, FVC, and the FEV1/FVC ratio (Li et al., 2021). This finding suggests that targeting multiple pathways with combination therapy may be a more effective approach for managing PH in patients who do not respond adequately to monotherapy (Ghofrani and Humbert, 2014; Pulido et al., 2013). Further research is needed to explore the potential benefits of dual PH-targeted therapies in COPD-associated PH.

This systematic review suggests that riociguat and sildenafil are unlikely to improve oxygenation in COPD patients with PH (Li et al., 2021; Vitulo et al., 2017; Karakitsos et al., 2013; Blanco et al., 2010; Blanco et al., 2013; Ghofrani et al., 2015; Pichl et al., 2019; Vonbank et al., 2003), with some studies even reporting a decline in PaO_2_ following sildenafil use (Blanco et al., 2010). This deterioration may be attributed to the loss of hypoxic vasoconstriction and resulting ventilation-perfusion mismatch (Alqarni et al., 2023b). Notably, a double-blind, placebo-controlled study involving 30 patients with severe COPD found that systemic administration of the endothelin receptor antagonist bosentan worsened hypoxemia, leading to a decline in arterial oxygen pressure and an increased alveolar-arterial gradient (Stolz et al., 2008). Since systemic administration of vasodilators can exacerbate ventilation-perfusion mismatch by relieving hypoxic vasoconstriction, further research is needed to assess the potential benefits of inhaled selective vasodilatory agents for COPD-associated PH. The inhalation route offers a targeted approach, delivering higher drug concentrations directly to the lungs, optimizing ventilation-perfusion matching, and potentially improving gas exchange efficiency (Kumbhare et al., 2022; Hill et al., 2015).

The amelioration of dyspnea and the optimization of the quality of life represent significant concerns for COPD patients with PH (Krompa and Marino, 2022; Sugarman and Weatherald, 2021). This review highlights favorable clinical outcomes associated with vasodilatory drugs in this population. Previous trials have demonstrated that sildenafil and tadalafil can alleviate dyspnea (Shrestha et al., 2017; Maron et al., 2022) and improve health-related quality of life (Maron et al., 2022). However, one study found no significant quality-of-life improvements in patients undergoing pulmonary rehabilitation while receiving sildenafil compared to those on placebo or standard COPD treatment (Blanco et al., 2013). These findings suggest that sildenafil may help relieve breathlessness and improve quality of life in mild to moderate cases but not in severe COPD-associated PH. Supporting this, a pilot double-blind, placebo-controlled trial reported improvements in both dyspnea and quality of life following sildenafil use (Vitulo et al., 2017). While PH-targeted therapies show promise, robust evidence remains limited due to small sample sizes and short trial durations.

### 4.1 Strengths and limitations

To the best of our knowledge, this systematic review is the first to summarize existing evidence regarding the effects of pharmacotherapeutic agents targeting the NO-sGC-cGMP pathway on clinical outcomes in PH patients due to COPD. This review encompasses randomized controlled trials, observational studies, and retrospective analyses evaluating various parameters, including pulmonary hemodynamics, exercise capacity, lung function, severity of dyspnea, quality of life, and oxygenation status. Despite its comprehensive approach, certain factors may limit the scope of this review. We were unable to conduct meta-analysis due to heterogeneity among the included studies in terms of disease severity, sample size, and clinical trial duration. In addition, some of studies included in this review had short follow-up periods which limits understanding of long-term efficacy and safety of NO-sGC-cGMP pathway-targeting agents for the management of PH due to COPD.

### 4.2 Future direction

Targeting the NO-sGC-cGMP pathway is currently used to treat different groups of PH, with sildenafil and tadalafil approved for Group 1, and riociguat approved for both Group 1 and Group 4 PH. It has recently received considerable attention as a promising intervention for those with COPD-associated PH. In addition, the findings of this systemic review suggest that it is likely that oral sildenafil, in particular, can improve quality of life, shortness of breath, and pulmonary hemodynamics. Given the fact that the only approved therapy for group 3 PH (for those with PH due to ILD) is administered through inhalation route (Nathan et al., 2021) and that there is currently a new ongoing study that aimed to assess the efficacy and safety of inhaled sGC stimulator on improving exercise capacity for adult patients with COPD-associated PH (Merck and Dohme, 2023), further investigations with standardizing methodologies and outcome measures are needed to explore the long-term efficacy and safety of NO-sGC-cGMP pathway-targeting agents, particularly inhaled therapies for the management of PH due to COPD.

## 5 Conclusion

The findings of this systematic review suggest that pharmacotherapeutic agents targeting the NO-sGC-cGMP pathway exhibit promising potential for improving pulmonary hemodynamics and mitigating dyspnea severity, potentially optimizing health-related quality of life in COPD patients with PH. However, notable discrepancy was observed in relation to the effects of these drugs on exercise capacity, lung function, and arterial oxygenation. This systematic review serves as a valuable resource for clinicians and researchers alike, offering insights into the current therapeutic landscape for PH and identifying potential avenues for further investigation.

## Data Availability

The original contributions presented in the study are included in the article/Supplementary Material, further inquiries can be directed to the corresponding author.
