# Supplementary material for: Drugs targeting the NO-sGC-cGMP pathway in the treatment of patients with COPD-associated pulmonary hypertension: a systematic review
Source: Front Pharmacol. 2025 Sep 5;16:1641932. doi: 10.3389/fphar.2025.1641932 (PMC12446836; doi:10.3389/fphar.2025.1641932)
Supplement: Supplementary file 1 [file Table1.docx]

Table 1. Summary of included studies.

| **Authors, years of publication & country** | | **Study design** | **COPD severity: *n* (male)** | | | **Medication, (dose, duration, groups)** | **Diagnostic test to confirm**  **PH, mean/median mPAP or PASP** | | **Baseline PVR, CO, and CI** | | **Outcome** | **Findings** | **Overall risk of bias assessment** |
| --- | --- | --- | --- | --- | --- | --- | --- | --- | --- | --- | --- | --- | --- |
| Patrizio Vitulo et al., 2017 [1]  Italy | | A 16-week, double-blind, multicenter, randomized, placebo- controlled trial | GOLD 2 and 3: 28 (21)  Control group: GOLD 2 and 3: 10 (8)  Treated group: GOLD 2 and 3: 18 (13) | | | Oral sildenafil (20 mg) given 3 times a day for 16 weeks  Treated group: sildenafil  Control group: placebo | RHC was used to confirm diagnosis of PH, mean mPAP for both groups was 39 mmHg  Treated group: mean mPAP: 39 mmHg  Control group: mean mPAP: 39 mmHg | | PVR: N/A  CO: N/A  CI: N/A | | **Primary outcome:**   - Pulmonary hemodynamics compared with the baseline.   **Secondary outcome:**   - Changes from baseline of   exercise capacity, pulmonary function, oxygenation, dyspnea and quality of life | Primary outcomes:   - **Pulmonary hemodynamics**: significant differences in PVR were found between treated and control groups   - **Placebo group**: baseline vs follow up visit (mean ± SE of PVR 6.27 ± 0.79 WU vs 6.36 ± 0.79 WU)   - **Treated group**: baseline vs follow up visit (mean ± SE of PVR 7.01 ± 0.59 WU vs 5.72 ± 0.62 WU)   - Difference in change of PVR (95% CI): –1.38 (≤ –0.05 ) WU, p = 0.04   - Difference in change of total PVR (95% CI): –1.80 (≤ –0.21) WU, p = 0.03   Secondary outcomes:   - **Exercise capacity**: no significant differences were observed in 6MWT between treated and control groups. - **Pulmonary function**:   - **Spirometry parameters**: no significant differences were found in spirometry parameters compared with baseline.   - **DLCO %**: an increase in DLCO % was seen in the treated groups compared with control groups. Difference in change of DLCO% (95% CI): 5.53 (≥ 0.26)%, p = 0.04. - **Oxygenation**: no significant differences were observed in PaO_2_ between treated and control groups. - **Dyspnea:** dyspnea level (assessed by mMRC) was improved in the treated groups compared with control groups. Difference in change of mMRC score (95% CI): –0.60 (≥ –0.31), p = 0.03 - **Quality of life**: an improvement in quality of life (assessed by SF36 general health) was found in the treated groups compared with control groups. Difference in change of SF36 general health score (95% CI): 9.85 (≥ 0.78), p = 0.04 | Low |
| Ying Li et al., 2021 [2]  China | | Non-randomized controlled clinical trial. | GOLD N/A: 90 (47) | | | **Group A:**  Oral sildenafil (50 mg) given twice a day for 4 weeks +Oral bosentan (62.5 mg), both were given twice a day for 4 weeks  **Group B:**  Inhaled iloprost (5 g/dose)  +Oral bosentan (62.5 mg), both were given twice a day for 4 weeks | Doppler ultrasound was used to confirm diagnosis of PH | | PVR: N/A  CO: N/A  CI: N/A | | **Primary outcome:**   - Change in exercise capacity, pulmonary function, oxygenation, inflammatory mediators and cardiac function compared with the comparison. | Primary outcomes:   - **Exercise capacity:** 6MWD levels in group A (sildenafil) after one month of treatment were significantly higher than they were in group B (p < 0.05). - **Pulmonary function:** compared with B group (inhaled iloprost), improvement in FEV_1_, FVC, and FEV_1_/FVC ratio was observed in those who received sildenafil in group A. - **Oxygenation:** those in group A (sildenafil) showed an improvement in PaO_2_ level compared with group B (inhaled iloprost) (p < 0.05). - **Inflammatory mediators:** the inflammatory cytokine (Interleukin (IL)-17, IL-13, and C-reactive protein) levels were lower in group A (sildenafil) compared with B group (inhaled iloprost) ) (p < 0.05). - **Cardiac function:** right ventricular ejection fraction (RVEF) levels in group A (sildenafil) after one month of treatment were significantly higher than they were in group B (p < 0.05). | Low |
| Shrestha SK et al., 2017 [3]  Nepal | | Prospective randomized controlled trial conducted in two tertiary referral centers | GOLD 1-3: 61 (28) | | | **Group A Control:** standard treatment modalities for COPD    **Group B Treated:** Oral sildenafil (25 mg) given 3 times a day for 4 weeks with all the standard treatment modalities for COPD | Echocardiography was used to confirm diagnosis of PH  **Control group:** Baseline mean ± SD of PASP was 75.9 ± 17.89 mmHg  **Treated group:** Baseline mean +SD of PASP was 66.77 ± 11.61mmHg | | PVR: N/A  CO: N/A  CI: N/A | | **Primary outcome:**   - Pulmonary hemodynamics, and exercise capacity compared with the baseline   **Secondary outcome:**   - Changes from baseline of change in dyspnoea and severity of PH | Primary outcomes:   - **Pulmonary hemodynamics**: significant decrease in PASP was found between treated and control groups   - Difference of means (0 to 4 weeks) for control group was 9.87 ± 7.84 mmHg whereas difference of means (0 to 4 weeks) for the treated group was 5.93 ± 7.44 mmHg (p = 0.048). - **Exercise capacity**: significant improvement in 6MWT was found between treated and control groups   - Difference of means (0 to 4 weeks) for control group was 48.13 ± 25.79 m whereas difference of means (0 to 4 weeks) for the treated group was 32.59 ± 32.96 m (p = 0.047).   Secondary outcomes:   - **Dyspnoea**:   - Modified Borg scale: No statistical difference of means (0 to 4 weeks) was found between control group and treated group (1.20 ± 1.92 vs 1.55 ± 1.23; p = 0.401)   - mMRC grading: Statistical difference of means (0 to 4 weeks) was found between control and treated group (p = 0.037). - **WHO functional class**:   - WHO functional class: No statistical difference of means (0 to 4 weeks) was found between control group and treated group (p = 0.071). | High |
| Sharif-Kashani, B et al., 2014 [4]  Iran | | Prospective, randomized, open-label parallel group study | GOLD N/A: 40 (31) | | | **Group A Control:** amlodipine 2.5-7.5 mg once daily for 2 weeks    **Group B Treated:** Oral sildenafil (20-25 mg) given 2 times a day for 2 weeks | Echocardiography was used to confirm diagnosis of PH  **Control group:** Baseline mean ± SD of PASP was 58.0 ± 11.8 mmHg  **Treated group:** Baseline mean +SD of PASP was 63.0 ± 12.5 mmHg | | PVR: N/A  CO: N/A  CI: N/A | | **Primary outcome:**   - NT-proBNP levels compared with the baseline   **Secondary outcomes:**   - Changes from baseline of change in pulmonary hemodynamics | Primary outcomes:   - **NT-proBNP levels:** no statistical difference of means (0 to 2 weeks) was found between control group and treated group (p = 0.185)   Secondary outcomes:   - **Pulmonary hemodynamics**: no statistical difference of means (0 to 2 weeks) was found in PASP between control group and treated group (p = 0.164) | Low |
| Alexandra Pichl et al., 2019 [5]  Germany | | Retrospective analysis | GOLD 4: 7 (0) | | | **One group:** riociguat | RHC was used to confirm the diagnosis of PH, mean mPAP was 46 mmHg | | PVR: 681 ± 143 dyn·cm^−2^·s^−1^  CO: N/A  CI: 2.25 ± 0.16 mL·min^−1^·kg^−1^ | | **Primary outcome:**   - Pulmonary hemodynamics, and exercise capacity, pulmonary function and oxygenation compared with the baseline | Outcomes:   - **Pulmonary hemodynamics:** - **mPAP**: no significant differences were found between baseline mean ± SEM mPAP (46 ± 4  mmHg) and mean ± SEM mPAP after treatment  (38 ± 3  mmHg, p = 1.0). - **PVR**: significant differences were found between baseline mean ± SEM PVR (681 ± 143 dyn·cm−2·s−1) and mean ± SEM PVR after treatment  (389 ± 68 dyn·cm−2·s−1, p < 0.001). - **CI**: no significant differences were found between baseline mean ± SEM CI (2.25 ± 0.16 mL·min−1·kg−1) and mean ± SEM CI after treatment  (3.02 ± 0.20 mL·min−1·kg−1, p = 1.0). - **PAWP**: no significant differences were found between baseline mean ± SEM PAWP (11 ± 2  mmHg) and mean ± SEM PAWP after treatment  (11 ± 1  mmHg, p = 1.0). - **Exercise capacity:** no significant differences were found between baseline mean ± SEM 6MWT (269 ± 28 m and mean ± SEM 6MWT after treatment  (269 ± 56 m, p = 1.0). - **Pulmonary function:** - **FEV1**: no significant differences were found between baseline mean ± SEM FEV1 (46 ± 3 %) and mean ± SEM FEV1 after treatment  (48 ± 5 %, p = 0.998). - **DLCO:** no significant differences were found between baseline mean ± SEM DLCO (44 ± 5 %) and mean ± SEM DLCO after treatment  (43 ± 5 %, p = 0.999). - **Oxygenation:** no significant differences were observed between baseline mean ± SEM PaO_2_ (65 ± 4 mmHg) and mean ± SEM PaO_2_ after treatment  (75 ± 2 mmHg, p = 0.901). |  |
| Bradley A. Maron et al., 2022 [6]  United States | | Prospective, multicenter, placebo‐controlled randomized clinical trial | GOLD 1-4: 24 (24) | | | **Group A Control:** Placebo    **Group B Treated:** Oral tadalafil (40 mg) given once‐daily for 12 months | RHC was used to confirm the diagnosis of PH  **Control group:** Baseline median (IQR) mPAP was 33 (27–35)  **Treated group:** Baseline median (IQR) mPAP was 30 (27–34) | | PVR: N/A  CO: N/A  CI: N/A | | **Primary outcome:**   - Exercise capacity compared with the baseline at 12 months   **Secondary outcome:**   - Changes from baseline of change at 6 month in pulmonary haemodynamics, exercise capacity, dyspnea and health‐related quality of life | Primary outcomes:   - **Exercise capacity at 12 months:** no significant difference was observed between control group and treated group in median [IQR] difference of 6MWD from baseline at 12 months (21 [−9 to 40] vs. 17 [−1.7 to 49] m, p = 0.65).   Secondary outcomes:   - **Pulmonary haemodynamics:** - No significant difference was observed between the placebo and tadalafil treatment groups from baseline at 6 months in **mPAP** (0.0 [−3.0 to 5.0] vs. −1.0 [−2.0 to 0.0], p = 0.65), **PVR** (−0.3 [−0.7 to 0.2] vs. −0.5 [−2.1 to 0.4] WU, **cardiac output** (−0.3 [0.8 – 0.8] vs. 0.4 [−0.7 to 0.5] L/min, p = 0.68), and **PAWP** (−0.5 [−4.0 to 2.5] vs. 1.0 [−1.0 to 4.0] mmHg, p = 0.15). - **Exercise capacity at 6 months:** no significant difference was observed between control group and treated group in median [IQR] difference of 6‐MWD from baseline at 6 months in 6‐MWD (16 [−12 to – 42] vs. 4.9 [−23 to 45] m, p = 0.64). - **Dyspnea:** Tadalafil improved dyspnea significantly at 6 months (assessed by the UCSD SOBQ) compared to the placebo group (−4 [−14 to 7] vs. 12 [−2.0 to 17], p = 0.02) - **Quality of life:** compared to baseline, subjects who received tadalafil had within‐patient improvement in health‐related quality of life SGRQ total score (−8.7 [−17 to 2.8], p = 0.049) and the SGRQ impact score (−11.2 [−22.4 to 4.89], p = 0.029) at 6 months. | High |
| Dimitrios Karakitsos et al., 2013 [7]  Greece | | Prospective non-randomized, non-controlled, single arm interventional trial | GOLD 1-4: 12 (9) | | | Oral sildenafil (80 mg/day) given via a nasogastric tube for 3 days after the administration of dobutamine | RHC was used to confirm the diagnosis of PH  Baseline mean (95% CI) was 49 (44–53) | | PVR: N/A  CO: N/A  CI: N/A | | **Primary outcome:**   - Pulmonary haemodynamics and oxygenation | Outcomes   - **Pulmonary haemodynamics:** significant changes were found from baseline (expressed as % of change- baseline, post- sildenafil and end- of-study respectively).   - mPAP (-8%, -19%, -14%)   - PVR (-40%, -51%, -42%)   - CI (49%, 54%, 48%) - **Oxygenation:** significant changes were found from baseline (expressed as % of change- baseline, post- sildenafil and end- of-study respectively).   - PaO_2_/FiO_2_ (NS, 52%, 86%) | High |
| Khaled Alkhayat et al., 2016 [8]  Egypt | Prospective placebo-controlled trial | | | **Total:**  GOLD 1-4: 139 (106)  **Treated group:** GOLD 1-4: 69 (49)  **Control group:**  GOLD 1-4: 70 (57) | **Treated group:**  Oral Sildenafil (20mg) given 3 times a day for 12 weeks+ conventional therapy for COPD  **Control group:**  Placebo for same period as treated group +conventional therapy for COPD | | | Echocardiography was used to confirm the diagnosis of PH  **Control group:** Baseline mean ± SD of PAP was 56 ± 16 mmHg  **Treated group:** Baseline mean +SD of PAP was 45 ± 13 mmHg | PVR: N/A  CO: N/A  CI: N/A | **Primary outcome:**   - Exercise capacity   **Secondary outcome:**   - Pulmonary haemodynamics | | **Primary outcomes:**   - **Exercise capacity**: sildenafil significantly improved exercise capacity as compared with the placebo group at week 4, and this effect was maintained at weeks 8 and 12. - Difference in change in mean of 6MWT distance was 51m   **Secondary outcomes:**   - **Pulmonary haemodynamics**: sildenafil improved pulmonary hemodynamics presented in the reduction of mPAP. - **Placebo group:** difference in change of mPAP mean from baseline to week 12: 0.6 mmHg. - **Treated group**: difference in change of mPAP mean from baseline to week 12: –2.1 mmHg | **Low** |
| S. Alp et al., 2005 [9]  Germany | Prospective clinical trial | | | GOLD 3-4: 6 (4) | **One group:**   - 50 mg of Sildenafil was given orally twice daily for the following 3 months. | | | Echocardiography was used to confirm the diagnosis of PH | PVR: 373  CO: N/A  CI: N/A | **Primary outcomes:**   - Pulmonary haemodynamics - Exercise capacity | | - **Pulmonary haemodynamics:**    - mPAP: 24.6 ± 4.2 mmHg ( decreased from 30.2±5.5mmHg)   - PVR: to 264 ± 52 dyn s cm^-5^ (decreased from 401±108 dyn s cm^-5)^ - **Exercise capacity:** - Distance: 433 ± 52 m (increased from 351 ± 49 m) | **Low** |
| Isabel Blanco et al., 2010 [10]  Spain | Randomized, open label, with blind evaluation, dose comparison trial in two parallel groups. | | | GOLD 3 and 4: 20 (17) | - **Group A:** Patients assigned to oral sildenafil 20 mg (*n* = 11) given 2 times a day for 2 weeks - **Group B:** Patients assigned to oral sildenafil 40mg (*n* = 9) given 2 times a day for 2 weeks | | | - Doppler echocardiography (estimated systolic PAP > 40 mmHg) - Right heart catheterization (17 patients had mean PAP >20 mmHg at rest, 3 patients had mean PAP ≤20 mmHg at rest and all patients had mean PAP >30 mmHg during exercise) | PVR: 339 dyn.s.cm^-5^  CO: 4.90 L.min^-1^  CI: 2.72 L.min^-1^.m^-2^ | **Primary outcomes:**   - Sildenafil effect on pulmonary hemodynamics and gas exchange at rest. - Sildenafil effect on pulmonary hemodynamics and gas exchange during exercise. | | **Sildenafil effect on pulmonary hemodynamics and gas exchange at rest:**   - mPAP: 21 ±10 (decrease of 6 mmHg from mean baseline value) - PVR: 346 ± 191 (decrease of 110 dyn.s.cm^-5^ from mean baseline value). - CO: 0. 4.77 ± 95 (a slight decrease of 0.13 L.min^-1^ from mean baseline value) - No differences were observed between the 20 and 40 mg doses, although there was a trend to a greater increase in CO with the 40 mg dose. - PaO_2_: 58 ± 11 (decrease of 6 mmHg from mean baseline value). - FEV_1_: 0.83 ± 0.33 (slight decrease by 0.05L from mean baseline value). - FVC: 2.66 ± 0.85 (slight decrease by 0.14L from mean baseline value).   **Sildenafil effect on pulmonary hemodynamics and gas exchange during exercise:**   - mPAP: 56 ± 14 (decrease of 11 mmHg from mean baseline value) - PVR: 537 ± 220 (decrease of 136 dyn.s.cm^-5^ from mean baseline value). - CO: 8.68 ± 2.29 (a slight decrease of 0.32 L.min^-1^ from mean baseline value) - No differences were observed between the 20 and 40 mg doses. - PaO_2_: 57 ± 12 (decrease of 1 mmHg from mean baseline value). | **Low** |
| Isabel Blanco et al., 2013 [11]  Spain, Barcelona | Double blind, randomized, placebo-controlled trial | | | GOLD 3: 60 (54) | - 20 mg oral Sildenafil 3 times daily for 3 months combined with pulmonary rehabilitation. - Oral placebo 3 times daily for 3 months combined with pulmonary rehabilitation. | | | Right heart catheterization (mPAP ≥ 25 mmHg.) | PVR: N/A  CO: N/A  CI: N/A | **Primary outcome:**   - - The gain in the cycle endurance time at a constant work-rate   **Secondary outcomes:**   - - Performance and peak oxygen uptake in the incremental exercise test, 6-min walk distance and quality of life.   - Arterial oxygenation | | **Primary outcomes:**   - **The gain in the cycle endurance time at a constant work-rate:** - Cycle endurance time on Sildenafil: 149s (reduced from 268s) - Cycle endurance time on Placebo: 169s (reduced from 221s)   **Secondary outcomes:**   - **Performance and peak oxygen uptake in the incremental exercise test. 6-min walk distance and quality of life:** - No significant changes. - Oxygen consumption: 770 ml.min^-1^ (from 862 ml.min^-1^) on Sildenafil and 721 ml.min^-1^ (from 743 ml.min^-1^) on Placebo. - Maximal exercise tolerance: 41 W (from 45 W) on Sildenafil and 38 W (from 40 ml.min^-1^) on Placebo. - 6 min walk distance: 374 m (from 397 m) on Sildenafil and 369 m (from 390 ml.min^-1^) on Placebo. - **Arterial oxygenation:** - No significant effects noted. | **Low** |
| Hossein A. Ghofrani et al., 2014 [12]  Germany | Exploratory, nonrandomized, nonblinded, noncontrolled pilot study. | | | GOLD 2 and 3: 22 (11) | - A single dose of Riociguat (1 (*n*=10) or 2.5 mg (*n*=12)), previously shown to be well tolerated) in the morning of each of two separate assessment days at least 48 hours apart. - In a subset of patients (*n* = 8), Riociguat administration was preceded by short-term administration of iNO 20ppm | | | Cardiac catheterization (mPAP ≥ 23 mmHg) | PVR: 353 dyn.s.cm^-5^  CO: 4.63 L.min^-1^  CI: N/A | - Hemodynamic parameters - Lung function and gas exchange | | **Hemodynamic Parameters: postbaseline effect (mean** ± SD, p value)   - **mPAP**: - 1mg: -3.60 ± 3.41 , p = 0.0086 - 2.5mg: -4.83 ± 4.17, p = 0.0020 - iNO: -3.88 ± 2.90, p = 0.0069 - **PVR**: - 1mg: -58.32 ± 50.46, p = 0.0053 - 2.5mg: -123.8 ± 73.53, p = 0.0002 - iNO: -57.14 ± 78.64, p = 0.1029 - **CO**: - 1mg: 0.66 ± 0.48, p = 0.0018 - 2.5mg: 1.61± 1.49, p = 0.0049 - iNO: -0.03 ± 0.40, p = 0.8627 - **CI**: - 1mg**:** 0.35 ± 0.26, p = 0.0020 - 2.5mg: 0.89 ± 0.83, p = 0.0051 - iNO: -0.02 ± 0.20, p = 0.8136   **Lung function and gas exchange:**   - There was no change in lung function after administration of riociguat. - There were no clinically relevant changes in oxygenation (change after 2 hrs): - 72.7% of patients required supplemental oxygen. | **Low** |
| Sebastian Holverda et al., 2008. [13]  Amsterdam. | Prospective clinical trial | | | GOLD 2-4: 18 (11) | Oral single dose of 50mg Sildenafil and Placebo in different days | | | Right heart catheterization (at rest: mPAP ≥ 25 mmHg) and (during exercise: mPAP ≥ 30 mmHg) | PVR:   - At rest: 280 - During exercise: 319   CO:   - At rest: 5.5 - During exercise: 8.0   CI:   - At rest: N/A - During exercise: N/A | **Primary outcome:**   - Investigate the acute effects of Sildenafil on mPAP, PVR, and CO at rest and during exercise.   **Secondary outcome:**   - Assess whether a possible attenuation of right ventricular afterload by Sildenafil translates into an increase in maximal exercise capacity. | | **Primary outcome:**   - **Effects of Sildenafil on mPAP, PVR, and CO at rest and during exercise (mean ± SD):** - mPAP mmHg (at rest): lower mPAP from 23 ± 10 (baseline) to 20 ± 10 (after sildenafil use), p > 0.05. - mPAP mmHg (during exercise): lower mPAP from 35 ± 14 (baseline) to 30 ± 14 (after sildenafil use), p < 0.05. - PVR d.s./cm^5^ (at rest): lower PVR from 280 ± 180 (baseline) to 251 ± 217 (after sildenafil use), p > 0.05. - PVR d.s./cm^5^ (during exercise): lower PVR from 319 ± 209 (baseline) to 314 ± 288 (after sildenafil use), p > 0.05. - CO l/min (at rest): increased CO from 5.5 ± 1.0 (baseline) to 6.1 ± 1.7 (after sildenafil use), p > 0.05. - CO l/min (during exercise): unchamged CO from 8 ± 1.7 (baseline) to 8 ± 2.4 (after sildenafil use), p > 0.05.   **Secondary outcome:**   - **Maximal exercise test:** - Maximal workload (% pred): 49 ± 28 ± (no change from baseline) - Peak oxygen uptake (% pred): 11.2 ± 1.3 (no change from baseline) | **High** |
| K Vonbank et al., 2003. [14]  Vienna, Austria. | Randomised controlled prospective open study | | | GOLD 3 = 40 (27) | - Oxygen therapy alone (*n* = 20) for 3 months. - A combination of oxygen and NO(15-25 ppm with mean of 20ppm) (*n* = 20) for 3 months. | | | Right heart catheterization (mPAP ≥ 25 mmHg) | PVR =  - 259 dyne.s-^1^.cm^-5^ (oxygen alone)  - 276.9 dyne.s-^1^.cm^-5^ (oxygen + NO)  CO =  - 5.5 l/min (oxygen alone)  - 5.6 l/min (oxygen + NO)  CI =  - 2.7 l/min/m^2^ (oxygen alone)  - 2.7 l/min/m^2^ (oxygen + NO) | the influence of pulsed inhalation of NO in combination with oxygen on the PVR after 3 months treatment compared with inhaled oxygen alone. | | **The influence of pulsed inhalation of NO in combination with oxygen on the PVR index (PVRI) after 3 months treatment compared with inhaled oxygen alone:**  **Short term effect of iNO:** Inhaled NO and oxygen significantly decreased PVR, PVRI and mPAP and increased CO and CI in all patients.   - mPAP (mean ± SD): 23.1 ± 5.3 (decreased by 4 mmHg from baseline, p < 0.0001) - PVR (mean ± SD): 193 ± 78.7 (decreased by 83 dyne.s^-1^.cm^-5^ from baseline, p < 0.0001) - CI (mean ± SD): 3.0 ± 0.6 (increased by 0.4l l/min/m^2^ from baseline, p < 0.01). - CO (mean ± SD): 6.2 ± 1.4 (increased by 0.6 L/min from baseline, p <0.01)   **Long term effect of iNO (3 months):** oxygen and iNO caused a significant reduction in:   - mPAP (mean ± SD): 20.6 ± 4.9 (decreased by 7 mmHg from baseline, p <0.001) - PVR (mean ± SD): 173 ± 87.9 (decreased by 103.8 dyne.s^-1^.cm^-5^ from baseline, p <0.001) - CO (mean ± SD): 6.1 ± 1.0 (increased by 0.5 L/min from baseline, p <0.025) - CI (mean ± SD): 3.0 ± 0.4 (increased by 0.4l l/min/m^2^ from baseline, p =0.138). - PaO_2_ (mean ± SD): 10.1 ± 1.6 (decreased by 0.4 kPa from baseline, p =0.739). - FEV_1_ (mean ± SD): 0.4 ± 1.07 (decreased by 0.02 L from baseline, , p =0.742). - FEV_1_%FVC (mean ± SD): 13.3 ± 45.5 (decreased by 1% from baseline, , p =0.797). | **High** |

**Abbreviations:** PH: pulmonary hypertension; COPD: Chronic Obstructive Lung Disease; GOLD2: Global Initiative for Chronic Obstructive Lung Disease 2 (moderate COPD) GOLD3: Global Initiative for Chronic Obstructive Lung Disease 2 (severe COPD); PGI_2_: prostaglandin I_2_; mPAP: mean pulmonary artery pressure; RHC: right heart catheterization; ABG: arterial blood gas; WHO-FC: World Health Organization functional class; PaO_2_: partial pressure of oxygen; 6MWT: 6-minute walk test; PFT: pulmonary function tests; SGRQ: St George's respiratory questionnaire; FEV_1_: forced expiratory volume in one second; FVC: forced vital capacity; VE: minute ventilation; DLCO: diffusing capacity of the lungs for carbon monoxide; QOL: quality of life; PaCO2: partial pressure of carbon dioxide in arterial blood; SaO_2_: oxygen saturation of arterial blood; D_A–a_ O_2_: alveolar-arterial oxygen gradient; VE/VO_2_: ventilatory equivalent for oxygen; VE/VCO_2_: ventilatory equivalent for carbon dioxide; Qs/Qt: pulmonary shunt fraction; PVR: pulmonary vascular resistance; PAWP: pulmonary arterial wedge pressure; RAP: right atrial pressure; CIx: cardiac index; CO: cardiac output; MAP: mean arterial pressure; SD: standard deviat

1. Vitulo, P., et al., *Sildenafil in severe pulmonary hypertension associated with chronic obstructive pulmonary disease: A randomized controlled multicenter clinical trial.* J Heart Lung Transplant, 2017. **36**(2): p. 166-174.

2. Li, Y., Y. Wang, and S. Liu, *Bosentan combined with sildenafil in the treatment of COPD patients with pulmonary arterial hypertension.* Am J Transl Res, 2021. **13**(10): p. 11522-11530.

3. Shrestha, S.K., et al., *Effect of Sildenafil Citrate on Pulmonary Arterial Systolic Pressure and Sub-maximal Exercise Capacity in Chronic Obstructive Pulmonary Disease.* Kathmandu Univ Med J (KUMJ), 2017. **15**(60): p. 271-278.

4. Sharif-Kashani, B., et al., *The Effect of Amlodipine and Sildenafil on the NT-ProBNP Level of Patients with COPD-Induced Pulmonary Hypertension.* Iran J Pharm Res, 2014. **13**(Suppl): p. 161-8.

5. Pichl, A., et al., *Riociguat for treatment of pulmonary hypertension in COPD: a translational study.* Eur Respir J, 2019. **53**(6).

6. Maron, B.A., et al., *Tadalafil for veterans with chronic obstructive pulmonary disease-pulmonary hypertension: A multicenter, placebo-controlled randomized trial.* Pulm Circ, 2022. **12**(1): p. e12043.

7. Karakitsos, D., et al., *Acute effect of sildenafil on central hemodynamics in mechanically ventilated patients with WHO group III pulmonary hypertension and right ventricular failure necessitating administration of dobutamine.* Int J Cardiol, 2013. **167**(3): p. 848-54.

8. Alkhayat, K. and M. Eid, *Sildenafil citrate therapy for secondary pulmonary arterial hypertension due to chronic obstructive lung disease.* Egyptian Journal of Chest Diseases and Tuberculosis, 2016. **65**: p. 805–809.

9. Alp, S., et al., *Sildenafil improves hemodynamic parameters in COPD--an investigation of six patients.* Pulm Pharmacol Ther, 2006. **19**(6): p. 386-90.

10. Blanco, I., et al., *Hemodynamic and gas exchange effects of sildenafil in patients with chronic obstructive pulmonary disease and pulmonary hypertension.* Am J Respir Crit Care Med, 2010. **181**(3): p. 270-8.

11. Blanco, I., et al., *Sildenafil to improve respiratory rehabilitation outcomes in COPD: a controlled trial.* European Respiratory Journal, 2013. **42**(4): p. 982-992.

12. Ghofrani, H.A., et al., *Acute effects of riociguat in borderline or manifest pulmonary hypertension associated with chronic obstructive pulmonary disease.* Pulmonary circulation, 2015. **5**(2): p. 296-304.

13. Holverda, S., et al., *Acute effects of sildenafil on exercise pulmonary hemodynamics and capacity in patients with COPD.* Pulmonary pharmacology & therapeutics, 2008. **21**(3): p. 558-564.

14. Vonbank, K., et al., *Controlled prospective randomised trial on the effects on pulmonary haemodynamics of the ambulatory long term use of nitric oxide and oxygen in patients with severe COPD.* Thorax, 2003. **58**(4): p. 289-293.
