# Supplementary material for: Drugs targeting the NO-sGC-cGMP pathway in the treatment of patients with COPD-associated pulmonary hypertension: a systematic review
Source: Front Pharmacol. 2025 Sep 5;16:1641932. doi: 10.3389/fphar.2025.1641932 (PMC12446836; doi:10.3389/fphar.2025.1641932)
Supplement: Supplementary file 2 [file Supplementaryfile1.pdf]

Supplementary Material

Drugs targeting the NO-sGC-cGMP pathway in the treatment of patients with COPD-associated pulmonary hypertension: a systematic review

Abdullah A. Alqarni[1,2*], Sara A. Alghamdi[3], Abdulelah M. Aldhahir[4], Jaber S. Alqahtani[5], Rayan A. Siraj[6], Ahmed H. Alasimi[7], Heba M. Bintalib[8,9], Abdulkareem A. AlGarni[10,11], Mansour Majrshi[12,13,2], Adel M. Alshabasy[14], Salma AlBahrani[15, 16] and Hassan Alwafi[17]

[1]Department of Respiratory Therapy, Faculty of Medical Rehabilitation Sciences, King Abdulaziz University, Jeddah, Saudi Arabia
[2]Respiratory Therapy Unit, King Abdulaziz University Hospital, Jeddah, Saudi Arabia
[3]Respiratory Care Department, AlSalama Hospital, Jeddah, Saudi Arabia
[4]Respiratory Therapy Program, Department of Nursing, College of Nursing and Health Sciences, Jazan University, Jazan, Saudi Arabia
[5]Department of Respiratory Care, Prince Sultan Military College of Health Sciences, Dammam, Saudi Arabia
[6]Department of Respiratory Care, College of Applied Medical Sciences, King Faisal University, Al Ahsa, Saudi Arabia
[7]Department of Respiratory Therapy, Georgia State University, Atlanta, GA, USA
[8]Department of Respiratory Care, King Saud bin Abdulaziz University for Health Sciences, Jeddah, Saudi Arabia
[9]King Abdullah International Medical Research Centre, Jeddah, Saudi Arabia
[10]King Abdulaziz Hospital, The Ministry of National Guard Health Affairs, Al Ahsa, Saudi Arabia
[11]King Saud bin Abdulaziz University for Health Sciences, College of Applied Medical Sciences, Al Ahsa, Saudi Arabia
[12]National Heart and Lung Institute, Imperial College London, London, UK.
[13]Respiratory Medicine, Royal Brompton Hospital, London, UK
[14]Anesthesia and Critical Care Department, King Abdulaziz University Hospital, Jeddah, Saudi Arabia
[15]Infectious Disease Unit, Specialty Internal Medicine, King Fahd Military Medical Complex, Dhahran, Saudi Arabia
[16]College of Medicine, Imam Abdulrahaman Bin Faisal University, Dammam, Saudi Arabia[17]Department of Clinical Pharmacology and Toxicology, Faculty of Medicine, Umm Al-Qura University, Makkah, Saudi Arabia

*** Correspondence:**
Abdullah A. Alqarni; aaalqarni1@kau.edu.sa

Keywords: pulmonary hypertension, COPD, nitric oxide pathway, Group 3 PH, soluble guanylate cyclase stimulators, sGC stimulators, sildenafil, PDE5 inhibitors, phosphodiesterase-5 (PDE5) inhibitors, tadalafil

Appendices

Search strategy for the systematic review

Table S1: Search strategy in Embase, Medline, Cochrane and Scopus

Search strategy in embase		
1	exp Lung Diseases, Obstructive/	466843
2	(chronic adj2 (air* adj2 obstruct*)).fx,kf,tw.	3437
3	((lung* or pulmon* or respirat* or bronchopulmon*) adj3 obstruct*).fx,kf,tw.	117028
4	((centriacinar* or centrilobular* or focal or panacinar* or panlobular* or pulmonar*) adj2 emphysem*).fx,kf,tw.	9132
5	exp Bronchitis/	81005
6	"bronchit* ".ab,fx,kf,ti,tw.	39118
7	(COAD or COBD or COPD).fx,kf,tw.	113253
8	exp Hypoxia/	163828
9	hypoxia.fx,kf,tw.	200650
10	(oxygen adj4 (lack* or deprivation*)).fx,kf,tw.	12742
11	1 or 2 or 3 or 4 or 5 or 6 or 7	578842
12	8 or 9 or 10	262978
13	11 or 12	834603
14	exp Hypertension/	977517
15	(hypertension or (blood adj2 pressure*)).ab,fx,kf,ti,tw.	1112864
16	14 or 15	1496446
17	exp nitric oxide/	182809
18	"nitric oxide*".fx,kf,tw.	212757

19	(genosyl or inomax or noxivent).fx,kf,tw.	70
20	nitrogen monoxide.fx,kf,tw.	917
21	(NO adj3 inhal*).fx,kf,tw.	3347
22	17 or 18 or 19 or 20 or 21	262407
23	exp guanylate cyclase activator/	77241
24	(guanylate cyclase or sGC or MK-5475 or riociguat).fx,kf,tw.	17433
25	exp sildenafil/	25012
26	exp tadalafil/	8528
27	(sildenafil or tadalafil or revatio or adcirca).fx,kf,tw.	15312
28	exp phosphodiesterase V inhibitor/	35579
29	(Phosphodiesterase-5 or PDE-5 or Phosphodiesterase-V or PDE-V).fx,kf,tw.	6328
30	((Phosphodiesterase or PDE) adj3 five).fx,kf,tw.	66
31	23 or 24	90374
32	25 or 26 or 27	29657
33	28 or 29 or 30	36648
34	31 or 32 or 33	124422
35	exp cyclic GMP/	35401
36	(cyclic guanosine monophosphate or cGMP).fx,kf,tw.	32889
37	35 or 36	46644
38	34 or 37	158382
39	13 and 16	77387
40	38 and 39	2636
41	limit 40 to english language	2473

Search strategy in Medline

1	exp Lung Diseases, Obstructive/	237609
2	(chronic adj2 (air* adj2 obstruct*)).fx,kf,tw.	2345
3	((lung* or pulmon* or respirat* or bronchopulmon*) adj3 obstruct*).fx,kf,tw.	77212
4	((centriacinar* or centrilobular* or focal or panacinar* or panlobular* or pulmonar*) adj2 emphysem*).fx,kf,tw.	7473
5	exp Bronchitis/	31545
6	"bronchit* ".ab,fx,kf,ti,tw.	25302
7	(COAD or COBD or COPD).fx,kf,tw.	60066
8	exp Hypoxia/	90274
9	hypoxia.fx,kf,tw.	139265
10	(oxygen adj4 (lack* or deprivation*)).fx,kf,tw.	9927
11	1 or 2 or 3 or 4 or 5 or 6 or 7	286758
12	8 or 9 or 10	186875
13	11 or 12	469635
14	exp Hypertension/	318299
15	(hypertension or (blood adj2 pressure*)).ab,fx,kf,ti,tw.	699870
16	14 or 15	764916
17	exp nitric oxide/	96460
18	"nitric oxide*".fx,kf,tw.	166458
19	(genosyl or inomax or noxivent).fx,kf,tw.	10
20	nitrogen monoxide.fx,kf,tw.	641
21	(NO adj3 inhal*).fx,kf,tw.	2400
22	17 or 18 or 19 or 20 or 21	185422
23	exp guanylate cyclase activator/	0
24	(guanylate cyclase or sGC or MK-5475 or riociguat).fx,kf,tw.	13143

25	exp sildenafil/	5882
26	exp tadalafil/	1705
27	(sildenafil or tadalafil or revatio or adcirca).fx,kf,tw.	9157
28	exp phosphodiesterase V inhibitor/	0
29	(Phosphodiesterase-5 or PDE-5 or Phosphodiesterase-V or PDE-V).fx,kf,tw.	4002
30	((Phosphodiesterase or PDE) adj3 five).fx,kf,tw.	51
31	23 or 24	13143
32	25 or 26 or 27	10308
33	28 or 29 or 30	4044
34	31 or 32 or 33	25042
35	exp cyclic GMP/	22778
36	(cyclic guanosine monophosphate or cGMP).fx,kf,tw.	25666
37	35 or 36	35166
38	34 or 37	53445
39	13 and 16	25522
40	38 and 39	514
41	limit 40 to english language	484

Search strategy in Scopus

(ALL (*obstructive AND lung AND diseases*) OR TITLE-ABS-KEY ((chronic AND air* AND obstruct*)) OR TITLE-ABS-KEY ((coad OR cobd OR copd)) OR ALL (((centriacinar* OR centrilobular* OR focal OR panacinar* OR panlobular* OR pulmonar*) emphysem*)) OR TITLE-ABS-KEY (bronchitis) OR TITLE-ABS-KEY ((oxygen AND lack* OR deprivation*)) AND ALL (hypertension) OR TITLE-ABS-KEY ((hypertension OR (*blood AND pressure*))) AND ALL (nitric AND oxide*) OR TITLE-ABS-KEY (genosyl OR inomax OR noxivent) OR TITLE-ABS-KEY (nitrogen AND monoxide) OR TITLE-ABS-KEY (inhal* AND nitric AND oxid*) OR ALL (guanylate AND cyclase AND activator) OR TITLE-ABS-KEY (guanylate AND cyclase OR sgc OR mk-5475 OR riociguat) OR TITLE-ABS-KEY (sildenafil OR tadalafil OR revatio OR adcirca) OR TITLE-ABS-KEY (phosphodiesterase AND v AND inhibitor) OR TITLE-ABS-KEY (phosphodiesterase-5 OR pde-5 OR phosphodiesterase-v OR pde-v) OR TITLE-ABS-KEY (phosphodiesterase OR pde) AND TITLE-ABS-KEY ((phosphodiesterase OR pde) five) OR

TITLE-ABS-KEY (cyclic AND guanosine AND monophosphate OR cgmp)) AND (LIMIT-TO (LANGUAGE , "English"))

Search strategy in Cochrane

ID Search Hits

#1 MeSH descriptor: [Lung Diseases, Obstructive] explode all trees 24926

#2 ((chronic NEXT(air* NEXT obstruct*))):ti,ab,kw OR (((lung* or pulmon* or respirat* or bronchopulmon*) NEXT obstruct*)):ti,ab,kw OR (((centriacinar* or centrilobular* or focal or panacinar* or panlobular* or pulmonar*) NEXT emphysem*)):ti,ab,kw (Word variations have been searched) 936

#3 MeSH descriptor: [Bronchitis] explode all trees 2325

#4 ((COAD or COBD or COPD)):ti,ab,kw (Word variations have been searched) 18948

#5 MeSH descriptor: [Hypoxia] explode all trees 3182

#6 ((oxygen NEXT (lack* or deprivation*))):ti,ab,kw (Word variations have been searched) 26

#7 #1 OR #2 OR #3 OR #4 OR #5 OR #6 40187

#8 MeSH descriptor: [Hypertension] explode all trees 26619

#9 ((hypertension or (blood NEXT pressure*))):ti,ab,kw (Word variations have been searched) 151215

#10 #8 OR #9 151215

#11 #7 AND 10 26369

#12 MeSH descriptor: [Nitric Oxide] explode all trees 2498

#13 (nitric oxide*):ti,ab,kw OR (genosyl or inomax or noxivent):ti,ab,kw OR (nitrogen monoxide):ti,ab,kw OR ((NO NEXT inhal*)):ti,ab,kw (Word variations have been searched) 9128

#14 (guanylate cyclase activator):ti,ab,kw OR ((guanylate cyclase or sGC or MK-5475 or riociguat)):ti,ab,kw (Word variations have been searched) 611

#15 MeSH descriptor: [Sildenafil Citrate] explode all trees 1091

#16 MeSH descriptor: [Tadalafil] explode all trees 538

#17 (sildenafil or tadalafil or revatio or adcirca):ti,ab,kw OR (Phosphodiesterase-5 or PDE-5 or Phosphodiesterase-V or PDE-V):ti,ab,kw OR (((Phosphodiesterase or PDE) NEXT five)):ti,ab,kw OR (phosphodiesterase V inhibitor):ti,ab,kw (Word variations have been searched) 3716

#18 #12 OR #13 OR #14 OR #15 or #16 OR #17 13070

#19 #11 AND #18 914

Risk Of Bias Assessment

Table S2: Summary of the Cochrane risk of bias in non-randomized studies assessment

Domain	Ying Li et al., 2021 [17]	Alexandra Pichl et al., 2019 [27]	Dimitrios Karakitsos et al., 2013 [21]	Khaled Alkhayat et al., 2016 [16]	S. Alp et al., 2005 [18]	Hossein A. Ghofrani et al., 2014 [26]	Sebastian Holverda et al., 2008 [19]
**Bias due to confounding**	Low	Low	Low	Low	Low	Low	Low
**Bias in selection of participants into the study**	Low	Low	High	Low	Low	Low	High
**Bias in classification of interventions**	Low	Low	High	Low	Low	Low	High
**Bias due to deviations from intended interventions**	Low	Low	Low	Low	Low	Low	Low
**Bias due to missing data**	Low	Low	Low	Low	Low	Low	Low
**Bias in measurement of outcomes**	Low	Low	Low	Low	Low	Low	Low
**Bias in selection of the reported result**	Low	Low	High	Low	Low	Low	High
**Overall bias**	Low	Low	High	Low	Low	Low	High

Table S2: Summary of the Cochrane risk of bias in non-randomized studies assessment

(visual "Risk of Bias" table)

Domain	Ying Li et al., 2021 [17]	Alexandra Pichl et al., 2019 [27]	Dimitrios Karakitsos et al., 2013 [21]	Khaled Alkhayat et al., 2016 [16]	S. Alp et al., 2005 [18]	Hossein A. Ghofrani et al., 2014 [26]	Sebastian Holverda et al., 2008 [19]
**Overall bias**	Low	Low	High	Low	Low	Low	High

Table S3: Summary of the revised Cochrane risk-of-bias tool for randomized trials

Domain	Patrizio Vitulo et al., 2017 [20]	Shrestha SK et al., 2017 [22]	Sharif-Kashani, B et al., 2014 [23]	Bradley A. Maron et al., 2022 [28]	Isabel Blanco et al., 2013 [25]	Isabel Blanco et al., 2010 [24]	K Vonbank et al., 2003 [29]
Risk of bias arising from the randomization process	Low	Low	Low	Low	Low	Low	Low
Risk of bias due to deviations from the intended interventions (effect of assignment to intervention)	Low	High	Low	High	Low	Low	High
Risk of bias due to deviations from the intended interventions (effect of adhering to intervention)	Low	High	Low	High	Low	Low	High
Risk of bias due to missing outcome data	Low	Low	Low	Low	Low	Low	Low
Risk of bias in measurement of the outcome	Low	Low	Low	Low	Low	Low	Low
Risk of bias in selection of the reported result	Low	Low	Low	Low	Low	Low	Low
**Overall bias**	Low	High	Low	High	Low	Low	High

Table S3: Summary of the revised Cochrane risk-of-bias tool for randomized trials

(visual "Risk of Bias" table)

Domain	Patrizio Vitulo et al., 2017 [20]	Shrestha SK et al., 2017 [22]	Sharif-Kashani, B et al., 2014 [23]	Bradley A. Maron et al., 2022 [28]	Isabel Blanco et al., 2013 [25]	Isabel Blanco et al., 2010 [24]	K Vonbank et al., 2003 [29]
**Overall bias**	Low	High	Low	High	Low	Low	High
